# Mapping the ‘funny bone’: neuroanatomical correlates of humor creativity in professional comedians

**DOI:** 10.1093/scan/nsab049

**Published:** 2021-04-28

**Authors:** Jacob Brawer, Ori Amir

**Affiliations:** Neuroscience, Pomona College, Claremont, CA 91711, USA; Psychological Science, Pomona College, Claremont, CA 91711, USA

**Keywords:** creativity, expertise, neuroanatomy, comedians, humor

## Abstract

What are the neuroanatomical correlates of expertise in a specific creative domain? Professional comedians, amateurs and controls underwent a T1 MRI anatomical scan. Measures of cortical surface area (gyrification and sulcal depth) and thickness were extracted for each participant. Compared to controls, professional comedians had a greater cortical surface area in the left inferior temporal gyrus, angular gyrus, precuneus and right medial prefrontal cortex. These regions have been previously implicated in abstract, divergent thinking and the default-mode network. The high degree of overlap between the regions of greater surface area in professional comedians with the regions showing greater activation in the same group during comedy improvisation in our previous work (particularly the temporal regions and angular gyrus) suggests that these regions may be specifically involved in humor creativity.

## Introduction

What are the neuroanatomical correlates of a sense of humor? Are there structural anatomical differences in the brains of individuals with an objectively greater sense of humor such as elite professional comedians? Are such neuroanatomical differences unique to comedic creativity or are they common correlates of all forms of creativity? While there have been studies exploring the neuroanatomical correlates of artificial measures of creativity and the brain anatomy of professional musicians, writers and visual artists relative to controls, there has not been any research exploring the brain structure of professional comedians (Schneider *et al.*, 2002; Chamberlain *et al.*, 2014; Chen *et al.*, 2020). In the present investigation, we compare the brain anatomy of elite professional comedians relative to amateurs and non-comedians, aiming to better understand the structural neural correlates of creative expertise.

Several studies have demonstrated links between learned or inherited skills and cortical anatomy, such as larger hippocampi in cab drivers who memorized the London map (Maguire *et al.*, 2006) and larger motor cortex sub-region dedicated to finger manipulation in string instrument players (Elbert *et al.*, 1995). The skills of creative artists such as comedians are likely the product of genetic predisposition as well as prolonged training (Greengross *et al.*, 2012) and as such are likely to leave their marks on the artists’ neuroanatomy.

Recently, a body of research has emerged exploring the functional neural correlates of creativity. Functional magnetic resonance imaging (fMRI) studies have focused on a range of different creative tasks, spanning from artificial creativity measurements, such as the ‘alternative uses task’ (Fink *et al.*, 2010), to those recognized as art forms, like musical improvisation (Bengtsson *et al.*, 2007; Villarreal *et al.*, 2013), creative writing (Shah *et al.*, 2013) and drawing (Schlegel *et al.*, 2015; De Pisapia *et al.*, 2016). A recent meta-analysis examined the domain-general patterning of creativity across three different artforms (musical, drawing and literary), demonstrating overlap in the pre-Supplementary Motor Area (SMA), left dorsolateral prefrontal cortex and right inferior frontal gyrus, furthering support for a centralized ‘general creativity’ network involved in a wide range of creative activities (Chen *et al.*, 2020). Another meta-analysis of 45 fMRI studies demonstrated regional activation in the bilateral occipital, parietal, frontal and temporal lobes across modalities of creativity (Boccia *et al.*, 2015). Verbal creativity was primarily localized to the left hemisphere—PFC, inferior frontal gyri, lingual gyrus, middle and superior temporal gyri, inferior parietal lobule, postcentral and supramarginal gyri, middle occipital gyrus and insula. Musical creativity and visuospatial creativity also had similar activation patterns but with additional domain-specific activation of the fusiform gyri and left precentral gyrus, respectively (Boccia *et al.*, 2015). Overall, the literature consistently demonstrates an interplay of the default mode network (DMN) and executive control network during tasks that involve creative improvisation (Boccia *et al.*, 2015; Tachibana *et al.*, 2019; Chen *et al.*, 2020).

Beaty *et al.* (2018) proposed a working model for creative thinking, involving three primary networks that play an interactive role in the generation of novel ideas (Beaty *et al.*, 2018): The DMN, which is implicated in mind-wandering and spontaneous thinking (Kucyi *et al.*, 2016), or the executive control network exerts focus and direction on the spontaneous thoughts generated by the DMN (Wei *et al.*, 2014), or; and, the salience network. comprising the bilateral insula and anterior cingulate cortex, The salience network, the authors suggested, serves as a toggle between idea generation and evaluation, intermittently feeding forward information from the DMN to the executive control network (Beaty *et al.*, 2015; Uddin, 2015). Beaty *et al.* (2018) study concluded that creative thinkers are better able to co-activate these networks, which are typically not activated simultaneously in less creative thinkers, providing a possible explanation for the ability to associate remote ideas (Beaty *et al.*, 2018).

In what ways might humor be unique as a form of creativity? A humorous stimulus often takes a verbal (e.g. joke) and/or visual (e.g. caricature) form (Watson *et al.*, 2007; Amir, 2016). The processing of a humorous stimulus, rather than its creation, is most commonly described in the cognitive literature as detection of incongruity followed by its resolution, which may lead to an amused response (Suls, 1972). fMRI studies of humor processing commonly find activation associated with the detection and resolution of the humorous stimuli in the bilateral inferior/medial/superior frontal gyri and inferior superior/medial/temporal gyri, as well as the temporoparietal junction (Bartolo *et al.*, 2006; Chan *et al.*, 2012, 2013; Amir *et al.*, 2013; Tian *et al.*, 2017). The amusement that may follow has been linked to activation in the ventromedial prefrontal cortex as well as bilateral activation of the amygdalae and parahippocampal gyri (Chan *et al.*, 2012, 2013). Humor comprehension requires cognitive processes similar to those involved in active creativity (Perchtold-Stefan *et al.*, 2020). However, nearly all fMRI studies of humor have explored the neural correlates of its passive comprehension, not its active creation.

In 2016, Amir and Biederman proposed humor creativity may provide an ideal case study for the neural correlates of creativity more generally since humorous statements can be generated rapidly, their quality (funniness) judged relatively easily, and their creators can be readily classified by expertise (i.e. professional, amateur and non-comedians). In their study, professional comedians, amateurs and controls were generating captions for NewYorker cartoons while undergoing fMRI. Comedic experience was correlated with a decreased activation in the medial prefrontal cortex (mPFC) and increased activation bilaterally in temporal association regions (TMP), in particular in the temporo-occipital junction—extending from the lateral occipital cortex to the angular gyrus. All participants, regardless of comedic expertise, exhibited greater TMP activation during humor improvisation than during a non-humorous creative task. The degree of TMP activation during the improvisation of humorous captions positively correlated with their funniness as judged by independent raters. Overall these results suggest an important role for TMP in humor creativity. To the extent that neuroanatomy reflects comedic skill, we hypothesized TMP would be more developed among professional comedians.

Compared with the functional imaging literature, research on the neuroanatomical correlates of creativity has been largely limited to artificial measures of creativity. In a 2013 review, Jung *et al.* (2013) summarized results from studies correlating two indices of creativity with structural data: the composite creativity index (CCI) and the creativity achievement questionnaire (CAQ). Voxel-based morphometry (VBM) was employed to estimate measures of cortical thickness and volume. Cortical thickness was positively correlated with higher CCI/CAQ scores in regions such as the striatum, precuneus, dorsolateral prefrontal cortex (Takeuchi *et al.*, 2010), superior parietal lobule (Gansler *et al.*, 2011), posterior cingulate and right angular gyrus (Jung *et al.*, 2010). A negative correlation with cortical thickness was found in the cuneus, angular gyrus, inferior parietal gyrus, fusiform gyrus and orbitofrontal gyrus (Jung *et al.*, 2010; Gansler *et al.*, 2011). Some of those regions overlap with the default-mode network (namely, precuneus, inferior parietal and medial/orbitofrontal gyri). More recently, trait creativity, as measured by the Williams Creativity Aptitude Test, has been linked with a higher gray matter volume in the right posterior temporal gyrus (Li *et al.*, 2015). Another study found a positive correlation between Creative Behavioral Inventory scores and the right premotor area (Zhu *et al.*, 2016). Shi *et al.* (2017), using the Creative Achievement Questionnaire, found that *artistic* creativity is associated with lower gray matter volume in the supplementary motor area and anterior cingulate cortex, and *scientific* creativity is associated with greater gray matter volume in the left middle frontal gyrus and left inferior occipital gyrus.

A consistent theme across both functional and neuroanatomical studies of creativity is the involvement of regions of the ‘DMN’, a network of regions typically activated during task-free mind wandering (Raichle, 2015) but which are also activated along with regions of the cognitive control and saliency networks during engagement in creative cognition in creative thinkers (Beaty *et al.*, 2018).

The current study aims to explore anatomical differences in the brains of professional comedians, amateur comedians and controls. Based on previous work demonstrating the importance of TMP in humor creativity (Amir and Biederman, 2016) and the dominance of the DMN more generally in creative cognition (Beaty *et al.*, 2018), we hypothesized that

- comedic skill would be positively correlated with an increased TMP cortical surface area.
- comedic skill would be positively correlated with an increased cortical surface area in regions of the DMN.

## Methods

Professional comedians, amateurs and controls underwent a high-resolution anatomical T1-MRI scan. Their cortical surfaces in selected cortical regions of interest (ROIs) were correlated with comedic expertise, controlling for demographic characteristics (age, gender and handedness). The study was approved by the Institutional Review Board of the University of Southern California (USC) and the participants all signed informed consent.

### Participants

Of the 41 participants scanned, 38 had anatomical scans of sufficient quality for the volumetric analysis. The 38 were categorized as follows:

A group of 12 professional comedians included one female and nine right-handed comedians, with a mean age of 35.75 years (ranged 26–47). Five were members of ‘Groundlings,’ a highly selective Los Angeles-based improv troupe (from which TV shows likes ‘Saturday Night Live’ are often populated), and seven were professional stand-up comedians, with significant TV accolades (such as Netflix or Comedy Central specials, Conan appearances).

A group of nine amateur comedians included two females and eight right-handed comedians, with a mean age of 27.2 years (ranged 20–33). This group was composed of individuals with several years of casual stand-up/improv experience but who lacked significant formal professional experience as comedians (i.e. they mostly performed at open mics or free shows and had little TV credit). Nevertheless, the individuals in this group were selected based on the (subjective) assessment that they had the potential of becoming successful professional comedians after accumulating additional experience.

A group of 17 controls included seven females and 13 right-handed comedians, with a mean age of 24.82 years (ranged 19–34). All participants in this group were honors students, graduate students or faculty of the University of Southern California and were chosen in order to match the high intellect observed in successful comedians according to Greengross *et al.* (2012).

### Data acquisition

All MRI images were scanned at USC’s Dana and David Dornsife Cognitive Neuroscience Imaging Center on a Siemens Trio 3T scanner with a standard 16-channel head coil. Each subject underwent a high-resolution T1-weighted structural scan using Magnetization Prepared Rapid Acquisition Gradient Echo (MPRAGE) sequence, with a repetition time (TR) = 1100 ms, 192 sagittal slices, 256 × 256 matrix size, 1 mm × 1 mm × 1 mm voxels (for full details see Amir and Biederman, 2016).

### Anatomical pre-processing pipeline

**Fig. 1.**
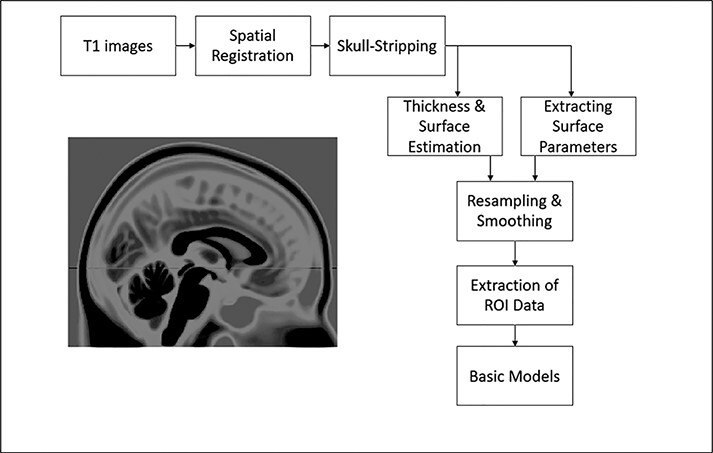
An overview of the preprocessing, extraction and analysis steps performed.

T1 images were pre-processed using MATLAB’s Statistical Parametric Mapping program (SPM12) with the computational anatomy toolbox (CAT12; Figure 1). First, T1 images were segmented and underwent standard CAT12 pre-processing procedures: affine regularization, full iterative SPM bias correction, skull stripping and spatial registration using optimized shooting registration that uses an adaptive threshold and a lower initial resolution, resulting in more accuracy and faster calculation time (Ashburner and Friston, 2011). A rigorous quality control protocol was followed in order to ensure the validity of the results. CAT12’s quality assurance framework allows for the assessment of Brain Web Phantom (BWP) noise, BWP bias and resolution and assigns scans an associated quality rating. T1 scans were also examined using a manual, visual quality assurance protocol as a final measure. Cortical surface area and cortical thickness estimation were then calculated and masked using the neuromorphometrics and lpba40 atlases for subsequent ROI analysis. Three different structural estimates were determined: cortical thickness, gyrification and sulcal depth. Cortical thickness was then resampled and smoothed with a 15 mm kernel, while cortical surface area measures (gyrification and sulcal depth) had a 20 mm kernel applied (standard kernel choices for the respective measures according to the CAT12 manual recommendations). From here, ROI values were determined (as detailed below).

**Fig. 2.**
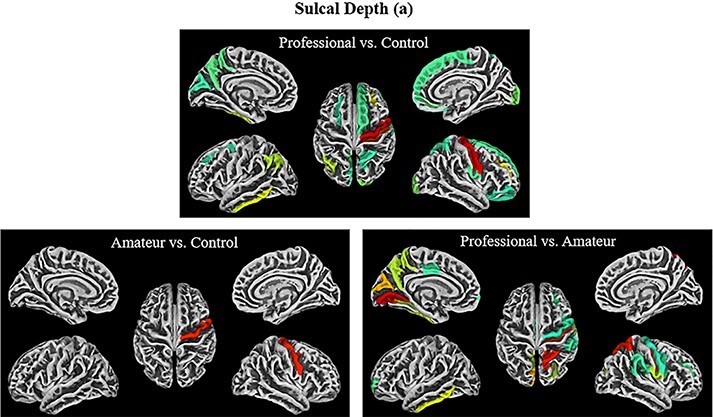
Sulcal depth contrasts (a2009s)**—**The visualizations above represent all ROIs from the a2009s atlas that showed significance on the measure of sulcus depth, under the following contrasts (from top left to bottom right): professional > control, amateur > control, professional > amateur (uncorrected). Note that the colors are only meant to delineate the ROIs and are not coding for any statistical measures.

### Surface-based morphometry

CAT12 utilizes an automated method for calculating cortical distance and central surface reconstruction—tissue segmentation is used to find the white matter distance, which then is projected to local maxima on the surface of the brain, identifying gray matter voxels and reporting distance. This projection-based method enables the estimation of partial volume information as well as sulcal blurring and sulcal asymmetries without explicit reconstruction (Dahnke *et al.*, 2013). Gyrification was calculated using absolute mean curvature, while sulcal depth was estimated using the square-transformed Euclidian distance between the central surface and the convex hull (Luders *et al.*, 2006a,b). CAT12 surface-based morphometry is also able to account for partial volume effects and also features a technique for topological correction that relies on spherical harmonics (Yotter *et al.*, 2011).

Surface-based methods were chosen as an anatomical measurement in this study for a multitude of reasons pertaining to the limitations of VBM. Several studies have demonstrated that VBM results are inherently interrelated with measures of cortical thickness, surface area and folding (Voets *et al.*, 2008; Hutton *et al.*, 2009; Righart *et al.*, 2017). It often becomes difficult to interpret VBM results, which are calculated using image intensities to estimate gray matter volume, when the underlying surface characteristics are the predominant cause of the observed cortical differences reported. For this reason, a surface-based approach, which individually targets the contribution of cortical thickness and measurements of folding, such as gyrification and sulcal depth, was chosen for the current study in order to explain anatomical differences in the brain in richer detail.

### Statistical design

All of the analyses described in this work are ROI analyses. We selected the ROIs following our two hypotheses based on the previous literature on the neural correlates of creativity in general and humor processing in particular, namely that TMP, as well as regions of the DMN, would display anatomical differences in relationship to comedic skill. A total of 11 bilateral ROIs were pre-selected and analyzed: angular gyri, precuneus, mPFC, temporal poles, inferior/superior temporal gyri, lateral occipital cortex, lingual gyri, middle anterior and posterior ventral cingulate cortex (isthmus) and supramarginal cortex.

For the sake of additional exploration, we repeated the same ROI analysis on every region in the native SPM atlases (DK40 and a2009s). We included all regions that showed significant differences between the groups in the tables and images, with the caveat that the reader is cautioned to interpret these results as exploratory, as the results are uncorrected. As mentioned in the results, this latter exploratory analysis only yielded one additional region, the cuneus, which we draw no conclusions about.

The three subject cohorts were entered as independent groups into a general linear model, regressing out sex, age and handedness as covariates of no interest. A series of *t*-contrasts were run post-hoc in order to compute the significance of surface-based measures differences between groups. η^2^ effect sizes were calculated for each contrast.

## Results

Measures of cortical thickness and surface area of professional comedians, amateurs and controls were compared in pre-selected ROIs which have been previously shown, in fMRI studies, to be involved in comedy improvisation specifically or consistently across different tasks involving creativity**—**in particular, regions of the DMN. A total of 11 bilateral ROIs were pre-selected and analyzed: angular gyri, precuneus, mPFC, temporal poles, inferior/superior temporal gyri, lateral occipital cortex, lingual gyri, middle anterior and posterior ventral cingulate cortex (isthmus), and supramarginal cortex. As detailed below, all but one of these pre-selected ROIs, the temporal poles, showed some significant anatomical difference between professional comedians and the rest.

**Fig. 3.**
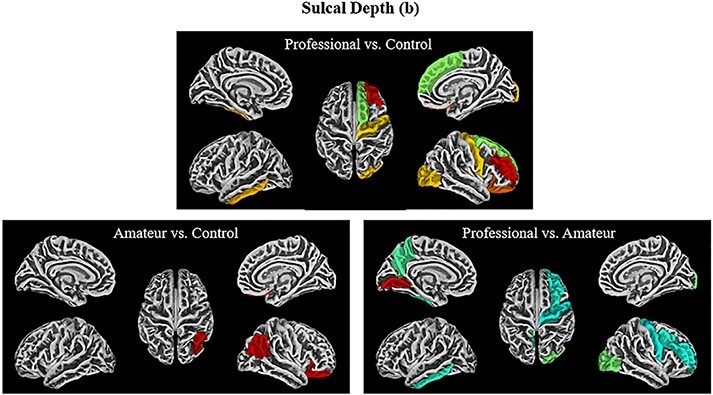
Sulcal depth contrasts (dk40)**—**The visualizations of all the ROIs from the DK40 atlas that demonstrated significance on the measure of sulcus depth with the following contrasts (from top left to bottom right): professional > control, amateur > control, professional > amateur (uncorrected). Note that the colors are only meant to delineate the ROIs and are not coding for any statistical measures.

**Fig. 4.**
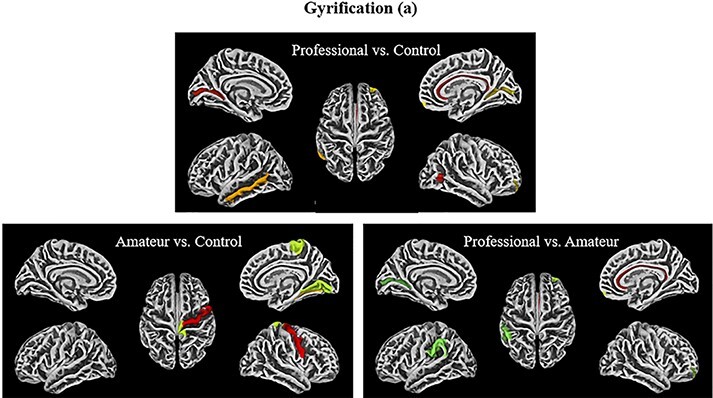
Gyrification contrasts (a2009s)**—**The visualizations above represent the ROIs from the a2009s atlas that demonstrated significantly greater gyrification for the following contrasts (from top-left to bottom right): professional > control, amateur > control, professional> amateur (uncorrected). Note that the colors are only meant to delineate the ROIs and are not coding for any statistical measures.

**Fig. 5.**
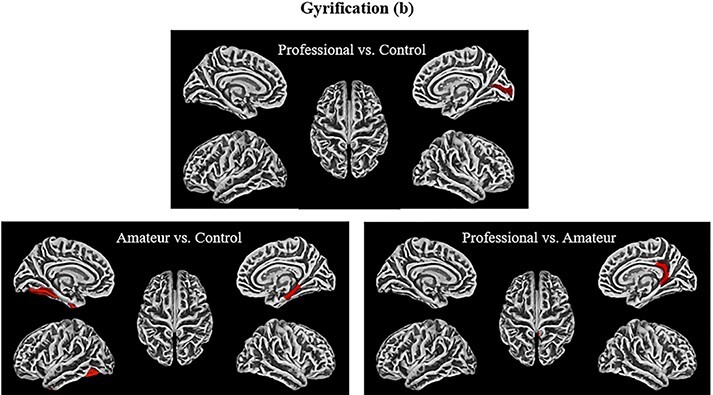
Gyrification contrasts (Dk40)**—**The visualizations above represent the ROIs from the DK40 atlas that demonstrated significantly greater gyrification for the following contrasts (from top-left to bottom right): professional > control, amateur > control, professional > amateur (uncorrected). Note that the colors are only meant to delineate the ROIs and are not coding for any statistical measures.

**Fig. 6.**
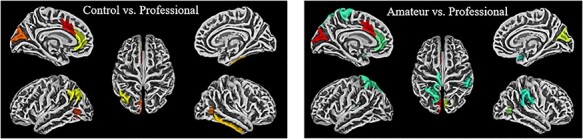
Cortical thickness contrasts (a2009s)—The visualizations above represent the ROIs from the a2009s that demonstrated significantly greater gyrification for the following contrasts: control > professional and amateurs > professional (uncorrected). Note that the colors are only meant to delineate the ROIs and are not coding for any statistical measures.

To avoid the multiple-comparisons problem (Thirion *et al.*, 2007), only pre-selected ROIs are discussed in the text and used as a basis for inference. However, as an additional exploratory analysis, we did run ROI analysis on each of the regions in two of SPM’s atlases in which only one additional region, the cuneus, was discovered to significantly anatomically differ among the groups. However, as we did not include the cuneus in our original hypothesis, and as this analysis was exploratory and uncorrected, we report the finding but draw no conclusions about the role of the cuneus. All significant ROIs are presented in Tables 1–7 and Figures 2–6.

**Table 1.** Sulcal depth—descriptive statistics (professional > control contrast)

*Professional vs control—SQ Sulc—a2009s*							
ROI	Prof mean	SE	Control mean	SE	*t*-value	*P*-value	Effect size
Left hemisphere							
Inferior temporal gyrus	2.14	0.07	1.91	0.05	2.32	0.01	0.38
Inferior angular gyrus	1.95	0.04	1.82	0.03	2.21	0.02	0.36
Precuneus	2.08	0.04	1.95	0.03	1.92	0.03	0.32
Cuneus	1.96	0.04	1.85	0.03	1.79	0.04	0.3
Right hemisphere							
Frontal middle sulcus	2.9	0.08	2.61	0.06	2.43	0.01	0.39
*Professional vs control—SQ Sulc—DK-40*							
Left hemisphere							
Inferior temporal	2.47	0.05	2.3	0.04	2.18	0.02	0.36
Right hemisphere							
Lateral occipital	1.89	0.03	1.8	0.02	2.13	0.02	0.35

**Table 2.** Sulcal depth—descriptive statistics (professional > amateur contrast)

*Professional vs amateur—SQ Sulc—a2009s*							
ROI	Prof mean	SE	Amat mean	SE	*t*-value	*P*-value	Effect size
Left hemisphere							
Medial temporal lingual gyrus	2.8	0.06	2.51	0.06	3.28	0.001	0.5
Cuneus	1.96	0.04	1.79	0.04	2.65	0.006	0.42
Inferior temporal gyrus	2.14	0.07	1.91	0.06	2.37	0.01	0.39
Precuneus	2.08	0.05	1.93	0.05	2.26	0.2	0.37
Right hemisphere							
Supramarginal gyrus	2.24	0.06	2.23	0.05	1.87	0.04	0.31
*Professional vs amateur—SQ Sulc—DK-40*							
Left hemisphere							
Lingual	3.42	0.05	3.16	0.05	3.55	0.0006	0.53
Precuneus	2.81	0.04	2.7	0.03	2.08	0.02	0.34
Inferior temporal	2.47	0.05	2.32	0.05	1.95	0.03	0.32
Right hemisphere							
Lateral occipital	1.89	0.03	1.79	0.03	2.23	0.02	0.37

**Table 3.** Gyrification—descriptive statistics (professional > control contrast)

Professional *vs* control—gyrification—a2009s							
Left hemisphere							
ROI	Prof mean	SE	Control mean	SE	*t*-value	*P*-value	Effect size
Middle temporal gyrus	27.15	0.36	26.73	0.28	1.9	0.03	0.32

**Table 4.** Gyrification—descriptive statistics (amateur > control contrast)

Amateur *vs* control—gyrification—a2009s							
Right hemisphere							
ROI	Control mean	SE	Amat mean	SE	*t*-value	*P*-value	Effect size
Midial temporal lingual gyrus	28.71	0.27	29.22	0.33	1.87	0.04	0.32

**Table 5.** Gyrification—descriptive statistics (professional > amateur contrast)

*Professional vs amateur—gyrification—a2009s*							
ROI	Prof mean	SE	Amat mean	SE	*t*-value	*P*-value	Effect size
Left hemisphere							
Supramarginal gyrus	27.34	0.42	26.25	0.39	1.84	0.04	0.31
*Professional vs amateur—gyrification—DK-40*							
Left hemisphere							
Isthmus cingulate gyrus	28.86	0.63	26.83	0.58	2.29	0.01	0.38

**Table 6.** Cortical thickness—descriptive statistics (control > professional contrast)

Control *vs* professional—cortical thickness—a2009s							
Left hemisphere							
ROI	Control mean	SE	Prof mean	SE	*t*-value	*P*-value	Effect size
Mid-cingulate anterior gyrus	3.12	0.04	2.96	0.05	2.43	0.01	0.39
Cuneus	2.12	0.03	2.01	0.03	2.13	0.02	0.35
Angular gyrus	2.94	0.04	2.82	0.05	1.73	0.04	0.29
Anterior cingulate gyrus	3.01	0.04	2.87	0.06	1.7	0.04	0.29

**Table 7.** Cortical thickness—descriptive statistics (amateur > professional contrast)

Amateur *vs* professional—cortical thickness—a2009s							
Left hemisphere							
ROI	Amat mean	SE	Prof mean	SE	*t*-value	*P*-value	Effect size
Cuneus	2.17	0.03	2.01	0.03	3.47	0.0007	0.52
Mid-cingulate anterior gyrus	3.19	0.05	2.95	0.05	3.42	0.0008	0.52
Anterior cingulate gyrus	3.04	0.05	2.99	0.06	2.08	0.02	0.35

Professional comedians compared to controls had greater sulcal depth in the following ROIs: in the left hemisphere: angular gyrus (*t* = 2.21, *P* < 0.02, η^2^ = 0.37), precuneus (*t* = 1.92, *P* < 0.032, η^2^ = 0.32) and inferior temporal gyrus (*t* = 2.33, *P* < 0.02, η^2^= 0.38; see Table 1, Figure 3). In the right hemisphere, the lateral occipital cortex (LOC; *t* = 2.13, *P* < 0.021, η^2^ = 0.35) and frontal middle gyrus extending to the mPFC (*t* = 2.42, *P* < 0.011, η^2^ = 0.39) (Table 2) (Figure 3) showed greater sulcal depth. The mPFC, along with the precuneus and angular gyrus, forms part of the DMN, implicated in spontaneous, creative thinking (Wei *et al.*, 2014). The angular gyrus, as well as the LOC, partially overlaps with the superior and inferior portions of the temporo-occipital junction (TOJ) respectively, regions that Amir and Biederman (2016) have shown have greater activation in professional comedians compared to controls when improvising humorous captions to cartoons. Within-subject, activation in the same ROI was positively correlated with the funniness of the improvised caption (Amir and Biederman, 2016). While the overlap with the functional ROI is not perfect, a recent review suggests that both Angular Gyrus (AG) and LOC are strongly linked anatomically and functionally with the temporo-occipital-parietal junction (TOPJ), and anatomical differences may be expressed metabolically (the measure employed by fMRI) in adjacent areas (Schurz *et al.*, 2017).

Contrasting professional and amateur comedians reve-aled greater sulcal depth in some of the same regions (Table 2; Figure 3), including the left precuneus (*t* = 2.26, *P* < 0.02, η^2^ = 0.37), and the left inferior temporal gyrus (*t* = 2.37, *P* < 0.012, η^2^ = 0.39). Other regions in the left hemisphere include the lingual gyrus (*t* = 3.28, *P* < 0.002, η^2^ = 0.50), which lies near the TOPJ and plays a role in divergent thinking, as well as the middle posterior cingulate cortex, a part of the DMN (Leech and Sharp, 2014; Zhang *et al.*, 2016). In the right hemisphere, greater sulcal depth was found in the supramarginal gyrus (*t* = 1.87, *P* < 0.04, η^2^ = 0.31), another region associated with the TOPJ according to a probabilistic atlasing study (Schurz *et al.*, 2017).

Exploring the same contrasts using gyrification measures reveals only a few significant ROIs. Professionals compared to controls have greater gyrification in the left medial temporal gyrus (*t* = 1.90, *P* < 0.033, η^2^ = 0.32) (Table 3; Figure 5). Amateurs displayed greater gyrification than controls in the right medial lingual gyrus (*t* = 1.88, *P* < 0.04, η^2^ = 0.31) (Table 4) (Figure 5). Professionals compared to amateurs showed greater gyrification in the left supramarginal gyrus (*t* = 1.84, *P* = 0.04, η^2^ = 0.32) (Table 5; Figure 4) and the right isthmus gyrus (*t* = 2.29, *P* < 0.02, η^2^ = 0.38) (Table 5; Figure 6), which connects the posterior cingulate gyrus to the hippocampus, a pathway involved in the DMN (Baker *et al.*, 2018).

When looking at the reversed contrasts, where control > professional, we see greater cortical thickness in some of the same default-mode regions that differed in sulcal depth and gyrification in the professional > control contrast. In the left hemisphere, the contrast was significant in the mid-anterior cingulate gyrus (*t* = 2.43, *P* = 0.01, η^2^ = 0.39), cuneus (*t* = 2.13, *P* = 0.02, η^2^ = 0.35), angular gyrus (*t* = 1.73, *P* = 0.04, η^2^ = 0.29) and anterior cingulate gyrus (*t* = 1.70, *P* = 0.04, η^2^ = 0.29; Table 6; Figure 6). The amateur > professional contrast revealed cortical thickness differences in three of the four same regions: cuneus (*t* = 3.47, *P* = 0.0007, η^2^ = 0.52), mid-anterior cingulate gyrus (*t* = 3.42, *P* = 0.0008, η^2^ = 0.52) and anterior cingulate gyrus (*t* = 2.08, *P* = 0.02, η^2^ = 0.35). These results suggest there is an inverse relationship between cortical thickness and measures of cortical surface area within our ROIs in the DMN and temporal lobes.

## Discussion

The goal of the current study was to explore whether comedic experience and skill are reflected in the structural anatomy of individuals’ brains. Surface-based morphometry was conducted on professional comedians, amateur comedians and controls, in order to extract potential anatomical differences in the cortical surface area (gyrification and sulcal depth) and cortical thickness in ROIs that previous functional and structural MRI research have linked with intelligence, creativity and more specifically, humor processing. These ROIs included several regions within the temporal lobes and DMN.

### Structural correlates of comedic expertise

Gyrification and sulcal depth both are measurements of cortical folding, which are commonly used proxy measures of its surface area. We found that greater comedic expertise was linked to a greater surface area in the left precuneus, angular gyrus and right medial PFC, key regions implicated in abstract, creative thinking necessary for the divergent thought process associated with humor generation (Amir and Biederman, 2016; Beaty, 2019). These findings generally confirmed our hypotheses. One study found that gyrification and sulcal depth were positively correlated with two measures of human intelligence, fluid and crystallized intelligence (Tadayon *et al.*, 2020). This is consistent with Greengross *et al.* (2012) work that demonstrated that professional comedians tend to score higher on measures of language-based intelligence. Since the control group was composed entirely of honor undergraduate students, graduate students and faculty, it is unlikely that the findings may be explained by professional comedians’ higher IQs. Rather, the difference should reflect the neuroanatomical correlates of a more specific skill that is more closely linked to comedic creativity.

### Cortical surface area *vs* thickness

In all of the ROIs examined, whenever group differences were found in the cortical surface area they always favored those with higher comedic skill, whereas, whenever cortical thickness differences were significant, cortical thickness was greater in the less comedically skilled. Often the same or partially overlapping ROIs displayed both differences. For example, professional comedians had a greater cortical surface area in the angular gyrus relative to controls, but controls had greater cortical thickness in the same area relative to the professionals. Recent work has demonstrated that there may be an inverse relationship between folding and cortical thickness; a phenomenon of ‘cortical stretching’ that increases the surface area, while limiting cortical thickness, consistent with our findings in our professional comedian subjects (Hogstrom *et al.*, 2013; Song *et al.*, 2015; Tadayon *et al.*, 2020). This cortical trade-off has been theorized to be related to an increase in intercolumnar connections horizontally, which allows for a greater allocation of energy for processes that occur within neighboring columns, and less vertical connectivity, resulting in less cortical thickness, possibly as a result of a process of cortical ‘pruning’ for increased efficiency. This increase in horizontal connection is associated with both fluid and crystallized intelligence (Song *et al.*, 2015; Tadayon *et al.*, 2020). In certain high-order semantic regions, a columnar structure exists in which neighboring columns may represent distinct, often remote, high-level concepts (Quiroga *et al.*, 2008). Horizontal connections between such columns may underlay the mechanism to remotely associate between these concepts, which form the basis for divergent, abstract thinking—that is foundational to creativity in general and comedic creativity in particular (Amir and Biederman, 2016).

### The ‘dose response’

Some fMRI studies have demonstrated a ‘dose response’, that is, an increase in the degree of a particular cognitive process is associated with an increase in activity in some of the cortical regions involved with that cognitive process. Examples include increased activity in visual regions with increased complexity and interpretability of the visual stimuli presented (Grill‐Spector *et al.*, 1998) and increased activation in humor-processing regions with the presentation of subjectively funnier jokes (Amir *et al.*, 2013). Amir and Biederman (2016) found that the same posterior temporal regions that were associated with humor improvisation were activated to a greater degree during the process of improvising the funnier jokes. Activity in the same posterior temporal regions during humor improvisation was greatest in professional comedians and greater in amateur comedians compared to controls (Amir and Biederman, 2016). The present investigation finds some evidence of an expertise ‘dose’ effect with respect to surface area. In particular, inferior temporal gyrus, precuneus and cuneus all demonstrate an increase in sulcal depth when comparing professionals to amateurs and professionals to controls, amateurs do show increased surface area in the same regions relative to controls but that last difference failed to reach statistical significance. This may be the result of insufficient statistical power or of the transition from amateur to professional being qualitatively different from that of a non-comedian to an amateur.

### **Structural correlates of creativity and the** DMN

Our findings are consistent with the broader literature on creativity and its links to the DMN. Previous work has identified the precuneus as a central ‘hub’ of the DMN, and strong associations have been drawn between precuneus function and creative ability (Kirk *et al.*, 2009; Utevsky *et al.*, 2014; Beaty *et al.*, 2015; Ogawa *et al.*, 2018). Our results provide neuroanatomical support to these previous findings demonstrating that the precuneus and other regions of the DMN have a greater surface area in subjects who demonstrated greater comedic creativity. Beaty (2019)’s review proposed that fundamental to creativity is a coupling between the DMN and the executive control network (ECN). The latter likely serves to direct the former to process task relevant ideas. The functional connectivity between the ECN and DMN is particularly apparent in the mPFC, precuneus and the anterior cingulate gyrus, all of which display a positive correlation between comedic skill and measures of surface area.

## Conclusion

We find expertise related to comedic performance to be associated with increased measures of cortical surface area in ROIs of the DMN as well as temporal cortex regions associated more specifically with humor creativity. Some of the anatomical effects display a comedic skill dose response, with the greatest cortical surface area measures found in professional comedians compared with both amateurs and controls. Measures of cortical surface area, in the present study, were often inversely related to measures of cortical thickness. The former measures may relate to a more richly interconnected columnar architecture in high-level semantic regions—which, in turn, may be the mechanism allowing professional comedians to link remote concepts and perspectives in a novel meaningful fashion, which we believe is a fundamental aspect of humor creativity.
